# Effect of Nitisinone on Aortic Stenosis Disease Progression in Patients With Alkaptonuria: An Analysis of the Suitability of Nitisinone in Alkaptonuria (SONIA) 2 Study

**DOI:** 10.7759/cureus.100123

**Published:** 2025-12-26

**Authors:** Callum Bruce, Priyanka Meenamkuzhy-Hariharan, Shahdat Hussain, Antonio Eleuteri, Lakshminarayan Ranganath, Michael Fisher, Richard Imrich, Jean-Baptiste Arnoux, Birgitta Olsson, Mattias Rudebeck

**Affiliations:** 1 Internal Medicine, Royal Liverpool University Hospital, Liverpool, GBR; 2 Internal Medicine, Countess of Chester Hospital, Chester, GBR; 3 Cardiology, Aintree University Hospital, Liverpool, GBR; 4 Medical Physics and Clinical Engineering, Royal Liverpool University Hospital, Liverpool, GBR; 5 Clinical Biochemistry and Metabolic Medicine, Royal Liverpool University Hospital, Liverpool, GBR; 6 Cardiology, Royal Liverpool University Hospital, Liverpool, GBR; 7 Institute of Clinical and Translational Research, Slovak Academy of Sciences, Bratislava, SVK; 8 Paediatric Metabolic Disease, Hôpital Necker-Enfants malades, Paris, FRA; 9 Pharmaceuticals, Gariguella AB, Ekerö, SWE; 10 Pharmaceuticals, OnPoint Science AB, Stockholm, SWE

**Keywords:** aortic stenosis (as), metabolic disease, nitisinone, transthoracic echocardiography, alkaptonuria

## Abstract

Background and aim

Alkaptonuria (AKU) is a rare metabolic disorder characterised by the accumulation of homogentisic acid (HGA). Deposition of HGA in the aortic valve leading to progressive aortic stenosis is a serious complication. Nitisinone has been shown to improve morbidity and slow disease progression in AKU, but the effects of this treatment on aortic stenosis progression have not yet been described. The objective of this study was to evaluate whether treatment with nitisinone attenuated the progression of aortic stenosis, as assessed by peak trans-aortic valve pressure (P_max_), in patients with AKU. This post-hoc analysis used longitudinal echocardiographic data from the Suitability of Nitisinone in Alkaptonuria (SONIA) 2, a four-year multicenter randomised controlled trial, to examine aortic stenosis disease progression.

Methods

Data were obtained from echocardiograms performed on 138 patients over 48 months of follow-up. A linear mixed-effects regression model was used to ascertain the difference in the maximal trans-aortic valve pressure gradient (P_max_) at baseline and 48 months between the treatment and control groups, adjusting for baseline P_max_ and other covariates.

Results

At baseline, 18/138 patients (13.0%) had aortic stenosis of varying degrees of severity, and 25/138 (18.1%) had aortic sclerosis. The difference in P_max_ between the control (N=69) and treatment (N=69) groups at baseline was 0.063 mmHg [95% CI: -0.054 mmHg to 0.18 mmHg) and did not reach statistical significance (p=0.23). At the end of the four-year treatment period, the difference in P_max_ was 0.10 mmHg (95% CI: -0.0007 mmHg to 0.20 mmHg) (p = 0.05), representing a modest but statistically significant between-group treatment effect.

Conclusion

Nitisinone may attenuate the progression of aortic stenosis in patients with AKU. Given the small absolute effect size and post-hoc nature of the analysis, these findings should be interpreted as exploratory and hypothesis-generating rather than clinically definitive. Additional research is needed to determine whether nitisinone produces clinically meaningful outcomes for aortic stenosis in this population.

## Introduction

Alkaptonuria (AKU), first described by Archibald Garrod in 1902 as an “inborn error of metabolism”, affects multiple organ systems [1]. Its prevalence is 1 in 500,000 in most non-consanguineous populations and may be diagnosed at birth due to dark urine [2], though most diagnoses occur in adulthood [3].

AKU is an autosomal recessive disorder resulting in loss of enzyme activity of homogentisate 1,2 dioxygenase, which is responsible for the metabolism of homogentisic acid (HGA), an intermediary product of tyrosine metabolism [3]. The subsequent accumulation and deposition of HGA, appearing as a brown-black pigment in connective tissue such as joint and spine cartilage, tendons, ligaments and heart valves, is known as ochronosis [3-6]. This destructive process manifests most prominently as spondyloarthropathy and cardiac valve disease, preferentially in the aortic valve with some involvement of the mitral and tricuspid valves [3-6]. The typical presentation of AKU is a triad of homogentisic aciduria, ochronosis and degenerative arthritis [7,8]. Previous studies have suggested a relationship between ochronosis and aortic stenosis (AS) [9,10]. The AS progresses gradually and silently, often going undetected until aortic valve surgery [7,11].

Nitisinone, an inhibitor of hydroxyphenylpyruvate dioxygenase activity, causing a 95% reduction in serum and urine HGA (surrogates for disease activity), has been approved as the first disease-modifying therapy in adult AKU by the European Medicines Agency [12-14]. The Suitability of Nitisinone in Alkaptonuria (SONIA) 2 study, a four-year randomised controlled trial, demonstrated that nitisinone 10 mg daily reduced urinary HGA and decreased progression of ochronosis and its clinical signs as represented by the cAKUSSI (clinical Alkaptonuria Severity Score Index) [13]. The SONIA 2 study did not primarily evaluate the effects of nitisinone on AS disease progression in AKU. However, a cohort analysis of 81 patients from the United Kingdom National Alkaptonuria Centre found that administration of nitisinone was accompanied by a significant reduction in AS disease progression, as there was evidence to suggest that the maximal trans-aortic velocity (V_max_) (an important parameter in classifying the grade of AS) change scores decreased over time following nitisinone therapy [2]. This previous cohort study provided supportive evidence linking ochronosis and aortic valve disease progression. Important differences between this observational cohort study and SONIA 2 include the lack of randomisation, smaller sample size and use of a lower nitisinone dose (2 mg). The main limitations of studies in AKU to date have been the relatively small numbers of patients, given the rarity of the disease, and the absence of randomised data to test whether nitisinone significantly reduces the natural progression of AS in AKU patients [13-15].

Despite multiple trials in the past, no medical therapy has convincingly been shown to slow haemodynamic progression and improve prognosis in patients with AS due to any aetiology. Lipid-lowering therapy, particularly statins, showed initial promise, but subsequent studies failed to demonstrate positive clinical outcomes (death or aortic valve replacement) [16].

Given the success of the SONIA 2 study in showing that nitisinone slowed disease progression, we hypothesised that nitisinone might mitigate the progression of AS in AKU patients. Accordingly, we undertook a post-hoc exploratory analysis of longitudinal echocardiographic data from SONIA 2 to evaluate whether treatment with nitisinone is associated with progression of aortic stenosis, using peak trans-aortic pressure gradient (P_max_) as the primary longitudinal measurement. Although AS progression was not a pre-specified outcome of SONIA 2, echocardiographic data were collected prospectively at all study visits, providing an opportunity for robust secondary analysis.

This article was previously presented as a poster at the European Society of Cardiology Congress on August 30, 2024. It was posted to the Authorea pre-print server on November 25, 2024.

## Materials and methods

Study design

The SONIA 2 study was a four-year, randomised, multicenter, open-label, evaluator-blinded, no-treatment-controlled, parallel-group study investigating the safety and efficacy of nitisinone in AKU patients. It recruited 138 patients with AKU across three investigational sites in Liverpool (UK), Piešťany (Slovakia) and Paris (France). Independent ethics committees at each centre approved the study. Patients above the age of 25 with confirmed AKU were included. AKU was verified by elevated urinary HGA excretion as well as a clinical manifestation, such as ochronosis or chronic back or joint pain. Patients were randomised 1:1 to either receive oral nitisinone 10 mg daily or no treatment (control). Urinary HGA levels and several manifestations of AKU were measured. There were no recognised cardiovascular contraindications to nitisinone at the time of the study. AS was not a pre-defined endpoint of SONIA 2. The present analysis is therefore a post-hoc exploratory evaluation of routinely collected echocardiographic data.

Echocardiographic acquisition

Patients attended their respective study sites at baseline, three, 12, 24, 36, and 48 months, henceforth called visits V1, V2, V3, V4, V5 and V6. During these visits, echocardiograms were performed on the patients, from which we extracted the raw data for analysis. In measuring the degree of AS, the maximal trans-aortic valve pressure gradient (P_max_) was derived from the maximum blood flow velocity (V_max_), measured by continuous wave Doppler, by the application of the modified Bernoulli equation (P_max_ = 4 x V_max_^2^). All echocardiograms were done and reported in accordance with standards defined by the British Society of Echocardiography's education committee [17]. Echocardiograms were performed locally at each site. Consistent with the evaluator-blind design of SONIA 2, image-based assessments were performed by assessors blinded to treatment. Although no formal echocardiographic core laboratory was employed for central re-analysis of images, all echocardiographic data underwent central verification by a senior investigator during data cleaning and analysis to identify internal inconsistencies or biologically implausible changes in P_max_ values or valve disease categorisation over time. Mild, moderate and severe aortic stenosis, as well as aortic sclerosis, were defined in accordance with British Society of Echocardiography guidance [18], as shown in Table 1. Aortic sclerosis may be defined on the basis of echocardiographic findings as thickening or calcification of the aortic valve leaflets without significant obstruction to blood flow, with a peak aortic velocity of less than 2.5 ms^-1^.

**Table 1:** Echocardiographic criteria for defining aortic sclerosis and the severity of aortic stenosis, as applied in the SONIA 2 study. SONIA, Suitability of Nitisinone in Alkaptonuria.

Category	Aortic sclerosis	Mild aortic stenosis	Moderate aortic stenosis	Severe aortic stenosis
Aortic valve appearance	Thickened or calcified leaflets without restricted motion	Thickened or calcified leaflets with mild restriction	Calcified leaflets with moderate restriction	Heavily calcified/immobile leaflets with significant restriction
Peak aortic jet velocity (ms^-1^)	< 2.5	2.6 - 2.9	3.0 - 4.0	More than or equal to 4.0
Mean trans-aortic pressure gradient (mmHg)	No significant gradient	< 20	20 - 39	More than or equal to 40
Aortic valve area (cm^2^)	Not applicable (leaflets open normally)	> 1.5	1.0 - 1.5	< 1.0

Statistical analysis

All analyses reported were post-hoc. Statistical analysis of raw SONIA 2 echocardiographic data was undertaken to assess AS disease progression. The primary objective was to evaluate the association between longitudinal P_max_ measurements and treatment, adjusting for demographic and clinical factors. P_max_ was selected as the primary measure of AS progression because it was consistently available across sites. Other indices (e.g. aortic valve area, mean gradient) were incomplete or inconsistently recorded and therefore unsuitable for longitudinal modelling.

A linear mixed-effects model was used. Fixed effects included treatment allocation, follow-up year, age, sex, and baseline P_max_, the latter modelled using a third-order polynomial to account for its potential non-linear association with subsequent P_max_. Relevant interaction terms (including treatment x follow-up year, baseline P_max_ x follow-up year, and age x sex) were incorporated to allow for potential differences in progression trajectories. A random intercept for each participant accounted for the intra-subject correlation of repeated measurements.

Residual error variance was stratified by study site, partially addressing centre-level differences in echocardiographic acquisition and measurement practice. By adjusting for baseline AS severity, demographic characteristics, treatment allocation and site-level variability, the model accounted for the principal sources of potential confounding inherent in the dataset. The statistician performing the analysis was blinded to treatment labels.

Missing P_max_ values at intermediate visits due to incomplete follow-up were handled through the mixed-model likelihood-based framework under a missing-at-random assumption, without imputation, thereby maximising data use without restricting analysis to complete cases. Patients with only a baseline measurement were excluded, whereas those with partial follow-up contributed all available observations. One outlier patient was excluded after Cook’s statistic identified implausible values.

Model assumptions, including the distribution of residuals and random effects and the variance structure, were assessed using standard diagnostics (residual plots, Q-Q plots and Cook’s statistics). Error variance was stratified by site to account for heterogeneity. Full details of the statistical model are provided in Appendix 1.

## Results

The SONIA 2 study enrolled 138 patients between May 7, 2014, and February 16, 2015, with 69 patients randomly assigned to each arm (nitisinone or no treatment). These 138 patients had yearly measurements of P_max_ from baseline to four years. Demographic data and baseline characteristics in the treatment and control groups were well balanced (Table 2). Of the 138 patients included, 134 (97%) were White. In the nitisinone group, 45/69 (65%) were male, whereas in the control group, 40/69 (58%) were male. The mean age was slightly lower in the control group (47.6 years) compared to the nitisinone group (49.0 years) (Table 2).

**Table 2:** Demographic data and baseline characteristics.

Variables	Control (N=69)	Nitisinone (N=69)	Total (N=138)
Mean age, years	47.6	49.0	48.3
Mean bodyweight, kg	74.1	74.8	74.4
Mean height, cm	167	166	167
Sex			
Female, n (%)	29 (42%)	24 (35%)	53 (38%)
Male, n (%)	40 (58%)	45 (65%)	85 (62%)
Race			
White, n (%)	67 (97%)	67 (97%)	134 (97%)
Black, n (%)	0	1 (1%)	1 (1%)
Asian, n(%)	2 (3%)	1 (1%)	3 (2%)
Number of patients per study centre			
Liverpool, UK; n (%)	21 (30%)	20 (29%)	41 (30%)
Piešťany, Slovakia; n (%)	32 (46%)	33 (48%)	65 (47%)
Paris, France; n (%)	16 (23%)	16 (23%)	32 (23%)

**Figure 1:**
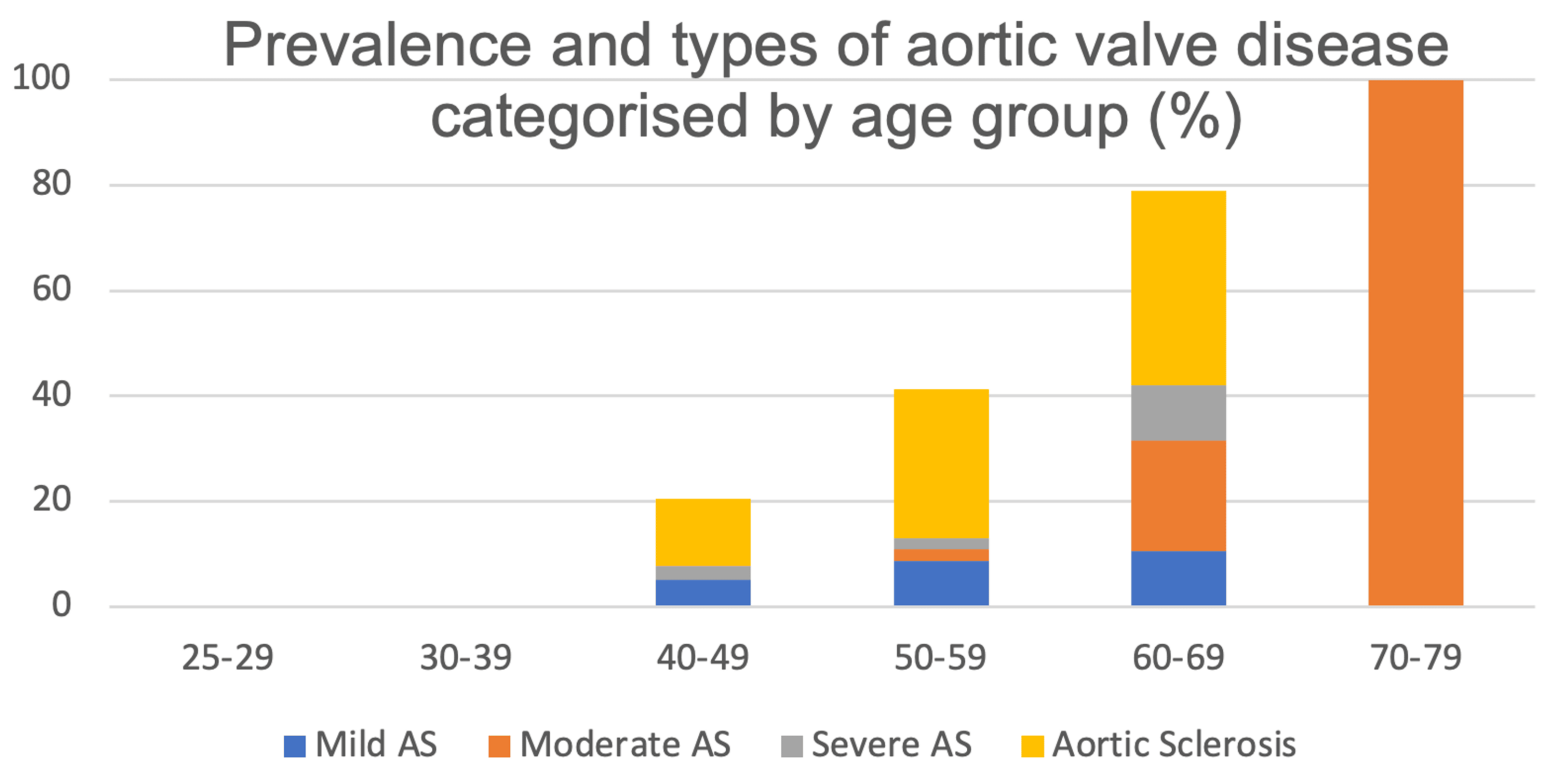
Prevalence and types of aortic valve disease by age group at baseline. AS, aortic stenosis.

Overall, 18/138 patients (13.0%) had AS at baseline, as classified by echocardiogram findings, with 8/18 (44.4%) having mild AS, 6/18(33.3%) having moderate AS and 4/18 (22.2%) having severe AS. 25/138 (18.1%) had sclerosis of the aortic valve. The prevalence of aortic valve disease increased with age (Figure 1).

**Figure 2:**
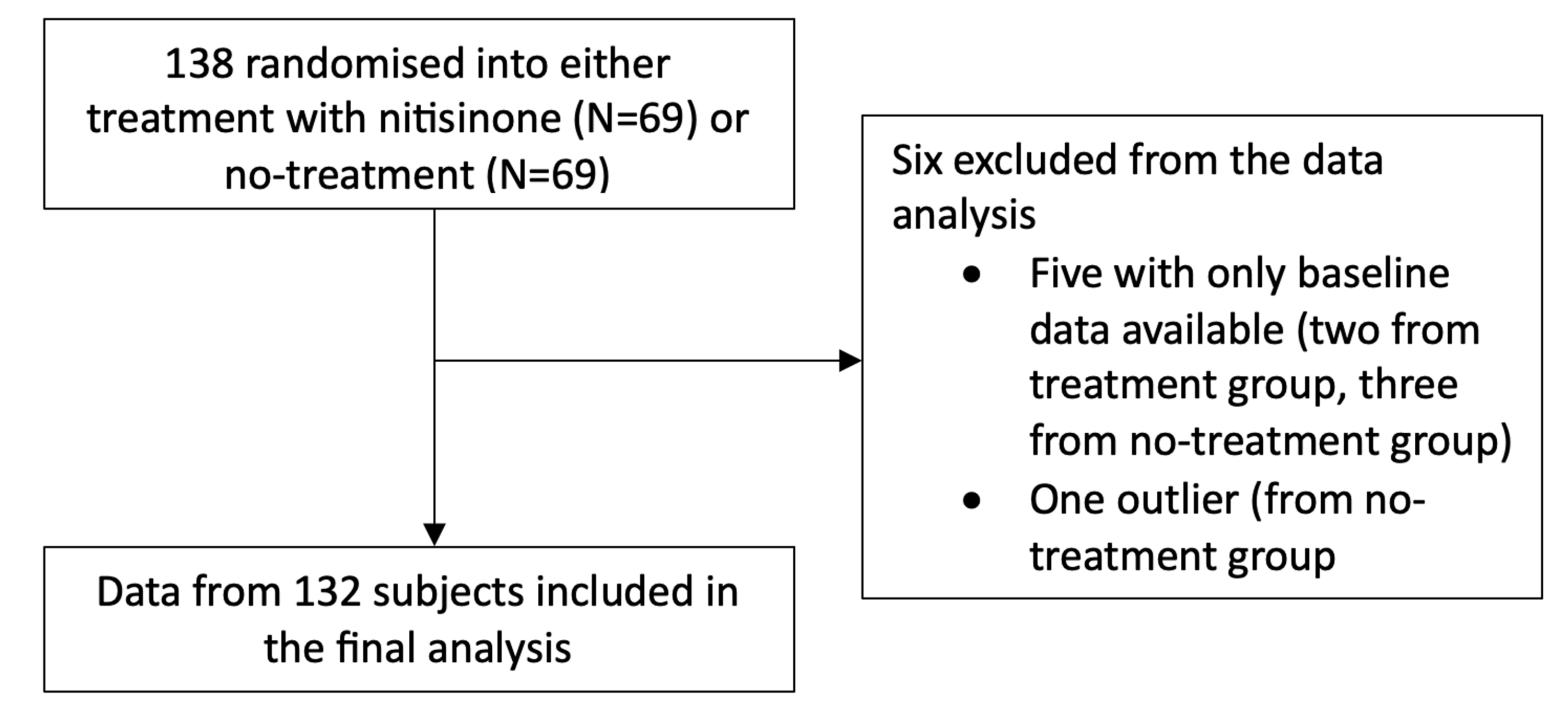
Flowchart showing subjects included in and excluded from the final analysis.

Our analysis looked specifically at AS disease progression as measured by P_max_. From scrutiny of the original data set of the SONIA 2 study, we were able to obtain a total of 613 completed longitudinal observations of 138 subjects across all three sites, with 96 of these patients having complete follow-up on every occasion from baseline to four years. The remaining 42 had more sporadic follow-up and did not present at every possible follow-up opportunity (see Appendix 2). Five patients with only a baseline measurement were excluded, and one patient was removed after Cook's statistic identified implausible and influential values. The final analysis, therefore, included 132 patients contributing 474 longitudinal observations (Figure 2).

**Figure 3:**
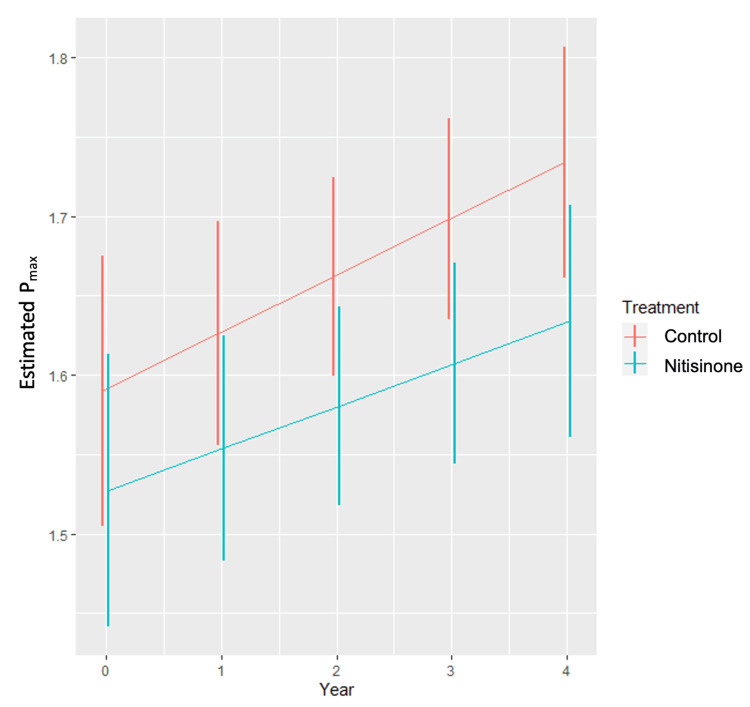
Graph representing the marginal estimated Pmax by year and treatment with 95% confidence intervals represented by the vertical overlapping lines.

The primary objective of the analysis was to assess whether P_max_ differed between groups over the four-year follow-up period. At baseline, the difference in P_max_ between the control and treatment groups was 0.063 mmHg (-0.054 mmHg, 0.18 mmHg) (p=0.23). At the end of the four-year treatment period, however, this difference was 0.10 mmHg (-0.00071 mmHg, 0.20 mmHg) (p=0.05). This represents a small but statistically significant difference favouring the nitisinone group at the end of follow-up. Estimated trajectories with 95% confidence intervals are shown in Figure 3. The mixed-effects model found no evidence of a statistically significant difference in the rate of progression between groups. The estimated difference between the rates of progression is 0.0093 mmHg/year (-0.020 mmHg/year, 0.039 mmHg/year) (p=0.53). There was no evidence of an effect of age (p=0.28), sex (p=0.62), nor interaction between age and sex (p=0.50). Overall, absolute mean changes in P_max_ over four years were small in both groups, consistent with a modest effect size despite statistical significance in the overall treatment term.

## Discussion

The analysis of the data from the SONIA 2 study showed a small but statistically significant difference in P_max_ between the nitisinone and no-treatment groups at the end of four years of follow-up. This represents an important addition to the literature, given the quality of evidence that randomised-controlled trials provide, as well as the statistically robust mixed-effects modelling applied. Our findings complement those of Ranganath et al., who, in a cohort study of 81 AKU patients assigned to 2 mg nitisinone or no treatment, demonstrated that nitisinone substantially reduced ochronosis burden and circulating HGA levels in AKU, although they reported minimal longitudinal change in V_max _[2]. Their study did not provide clear evidence of a significant treatment-associated reduction; however, their observations support the concept that the pathophysiological processes linked to ochronosis may be modifiable. It should be noted that their study was non-randomised with a less well-matched control group than SONIA 2, underscoring the relative robustness of our findings.

It is also important to address that the SONIA 2 study concluded that nitisinone did not significantly influence AS, which contrasts with the findings presented here. This discrepancy may be explained by our use of a more robust mixed-effects model, specifically designed for this measure, which increased statistical power by incorporating repeated measures. SONIA 2 compared only the mean final P_max_ between the treatment and control groups, whereas our analysis incorporated baseline P_max_ between groups and modelled change over time. Crucially, our findings do not indicate a statistically significant difference in the rate of progression, but rather a significant divergence in P_max _by year four, reflecting cumulative small differences over time. Specifically, the annual rate of increase in P_max_ was similar between groups, but the nitisinone group consistently exhibited slightly lower P_max _values across repeated measurements, leading to a statistically detectable separation at the end of follow-up. Because the model adjusted for baseline P_max_, the treatment groups were effectively compared as if they began at the same severity. Although the confidence intervals in Figure 3 overlap, this is expected with modest effect sizes; the statistical significance arises from the longitudinal divergence across repeated measures captured by the mixed-effects model, rather than from the separation of point estimates at individual visits. This subtle, reproducible pattern is biologically compatible with the expected effects of HGA suppression and reduced ochronosis.

The cardiovascular manifestations of AKU are characterised by ochronosis within cardiac connective tissues, including valves, endocardium, aortic intima and coronary arteries, with associated dystrophic calcification [9]. It is generally accepted that AS is the most common cardiac manifestation of AKU, and ochronotic deposition has indeed been found preferentially in areas of turbulent flow, including the coronary ostia and aortic valve leaflets [19]. Numerous surgical case reports have documented instances in which AKU has been discovered on the basis of findings during surgery, where significant ochronosis causing aortic stenosis has been seen [7,20]. A number of theories have been proposed regarding the pathogenesis of the valvular disease. It has been suggested that ochronotic pigmentation within cells found in cardiac tissue, including smooth muscle cells, macrophages and fibrocytes, may lead to extracellular ochronotic deposition, which may serve as a stimulus for dystrophic calcification through both inflammation and alteration of cartilage metabolism [21]. Interestingly, however, some authors have suggested that ochronosis is not responsible for functional derangement or the arteriosclerotic process itself, and appears in already diseased and calcified valves and atherosclerotic vessels [3,22,23]. This interpretation conflicts with the findings of Ranganath et al. and with our analysis [2]. Although exploratory, our data strengthen the hypothesis that metabolic suppression of HGA and ochronosis may influence valvular pathology over time.

The potential implications of nitisinone for patients with AKU in the context of AS are important, given that an increased prevalence of AS has been identified in patients with AKU compared to the general population. A case series of 76 AKU patients found an overall AS prevalence of 9% but in those aged 65 years and older, 50% had AS or had undergone valve replacement [9]. Another study of 16 patients found a significant burden of previously undiagnosed aortic valve disease in AKU patients, with 40% having aortic valve disease after the fifth decade of life and 50% after the sixth decade of life [10]. SONIA 2 also reported a prevalence of 13.8%, which increased with age. In general populations, AS is particularly uncommon in those aged under 65. A prospective population-based study of 3273 participants found prevalence to range from 0.2% in those aged 50-59 years to 9.8% in those aged 80-89 years [24]. It is therefore clear that AKU patients represent a significantly affected group.

Given the increased incidence of valvular disease in AKU patients with advancing age, there is an emerging consensus for echocardiographic screening after the age of 40 as well as assessment of coronary artery calcification using cardiac computed tomography in this patient group [7]. This would allow for early recognition of disease, with interventions aimed at reducing the likelihood of having to undergo major open-heart surgery, which is often challenging when operating on friable ochronotic valves where embolisation and subsequent stroke are a real risk [2]. Earlier identification would offer opportunities for patient education on symptoms and regular clinical review.

It should be acknowledged that although the difference in P_max_ at four years between the nitisinone and no-treatment groups was statistically significant, the magnitude of this difference is small and unlikely to be clinically meaningful at this stage. These findings should therefore be regarded as exploratory and hypothesis-generating rather than definite evidence of clinical benefit. Nonetheless, they provide a strong rationale for further research examining clinically meaningful endpoints such as symptoms, adverse cardiac events and attenuation of progression to severe disease, both within and beyond four years, in studies specifically designed and powered for valvular outcomes. Future studies should pre-specify AS progression as a primary or secondary endpoint and incorporate validated echocardiographic measures alongside surrogate biomarkers such as systemic HGA, ochronosis burden and imaging markers of calcification. Additional randomised-controlled trials with larger cohorts, longer follow-up and explicit focus on AS progression would be ideal, though the rarity of AKU poses significant practical challenges. Despite this, our findings are important and suggest that the timely initiation of nitisinone may reduce the incidence or delay the onset of severe aortic valvular disease, causing significant functional impairment.

This analysis has several limitations. P_max_, although derived from V_max_, which is the most reproducible Doppler measure, is still susceptible to technical variability such as imperfect transducer alignment [25]. The four-year follow-up is relatively short for assessing AS outcomes, although a between-group difference was still detectable. The sample size is small, reflecting the rarity of AKU, and only one functional outcome (P_max_) could be examined using SONIA 2 data. We were unable to assess clinical endpoints such as symptoms or adverse cardiac events, or to examine relationships between P_max_ progression and broader clinical severity measures such as the cAKUSSI score. Because AS progression was not a pre-specified endpoint in SONIA 2, this analysis is post-hoc and therefore more susceptible to bias than a prospectively defined comparison. Although the mixed-effects model adjusted for baseline P_max_ and handled incomplete follow-up under a missing-at-random assumption, non-random missingness cannot be excluded. Echocardiography was performed across three centres, and although site stratification was included, residual inter-site variability remains possible. Finally, unlike non-AKU AS trials that enrol only patients with established AS, relatively few participants in this dataset had AS at baseline, reducing statistical power; given the rarity of AKU, this is difficult to overcome.

Despite these limitations, our analysis provides valuable insights into how nitisinone may influence AS disease progression amongst a paucity of literature on this subject. Within AKU specifically, this may represent the first indication from randomised data that a pharmacological therapy could alter the trajectory of ochronotic aortic valve disease. This could have implications for future requirements for aortic valve surgery in affected patients.

## Conclusions

In summary, this post-hoc exploratory analysis of echocardiographic data from the SONIA 2 randomised controlled trial suggests that treatment with nitisinone may be associated with modest attenuation of cumulative differences in aortic valve disease progression in patients with alkaptonuria. Using longitudinal modelling of peak transaortic valve pressure (P_max_), we identified a small but statistically coherent divergence in valvular trajectories over four years, consistent with the expected effects of homogentisic acid suppression and reduced ochronosis. While the absolute magnitude of this difference is not clinically meaningful at this stage and should not be interpreted as evidence of clinical benefit, these findings provide a biologically plausible signal that pharmacological modification of valvular disease in AKU may be possible. Given the post-hoc nature of the analysis, the limited sample size, and the short duration of follow-up for a slowly progressive condition, the results should be regarded as hypothesis-generating. Further studies with longer follow-up, larger cohorts, and pre-specified valvular endpoints, including clinically meaningful outcomes, are warranted to determine whether early initiation of nitisinone can translate into tangible cardiovascular benefit in this rare disease population.

## Figures and Tables

**Figure 4:**
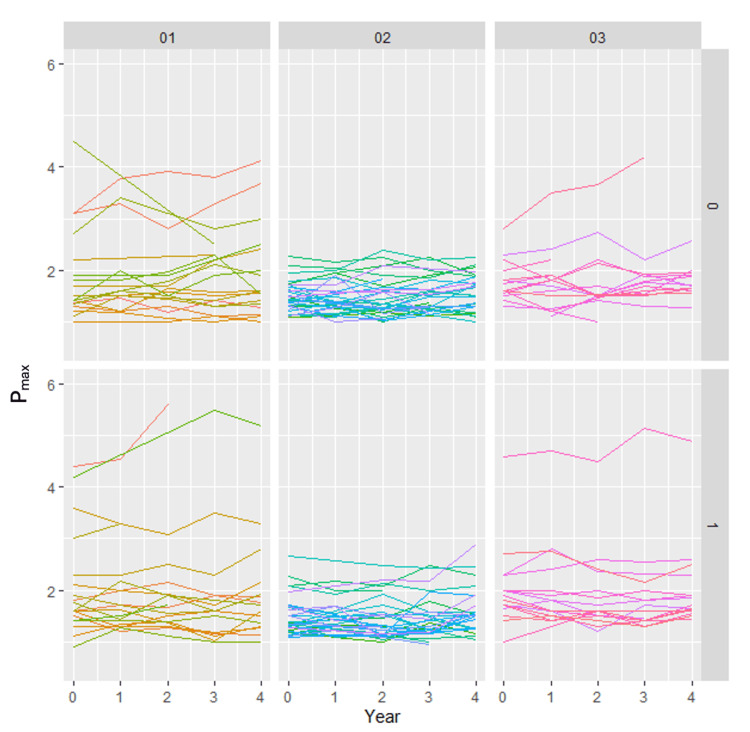
Yearly Pmax measurements by treatment (rows) and site (column).

## Appendices

Appendix 1

The linear mixed effects regression model can be written in matrix notation as:

y_i_=X_i_β+Z_i_b_i_+ϵ_i_

b_i_~N(0,σ_b_^2^), ϵ_i_~N(0,σ^2^δ_si_^2^)

where for the i^th^ subject:

-     y_i _is the vector of longitudinal aortic valve pressure (AVP) observations,

-     X_i _is the known fixed effects regressor matrix,

-     Z_i _is the known random effects regressor vector,

-     b_i _is the Gaussian random effect with variance,

-     ϵ_i _is the vector of Gaussian within-group errors with site-dependent variance,

-     β is the vector of fixed effects (coefficients of the model).

Baseline P_max_ was nonlinearly transformed via a 3rd order polynomial expansion to model potential nonlinearities.

The fixed effects regressor matrix represents the predictors available for each subject: age, sex, treatment group, follow-up years, nonlinear baseline P_max_, interaction between follow-up years and treatment group, interaction between nonlinear baseline P_max_, and interaction between age and sex. The random effects regressor matrix represents the subject-specific intercept of the trend of P_max_, and the within-group error term represents unexplained (by the model) variability in the observed data, stratified by site.

In Table 3 are reported the estimated subject-specific effects with attendant F statistics.

**Table 3:** F statistics for effects, including numerator (a) and denominator (b) degrees of freedom for the test and p-value.

Effect (subject-specific)	a	b	F(a,b)	p-value
Follow-up years	1	334	10.5	0.0013
Treatment	1	127	4.16	0.043
P_max_ at baseline (nonlinear 3^rd^ order polynomial expansion)	3	334	440	< 0.001
Age	1	127	1.17	0.28
Sex	1	127	0.25	0.62
Interaction between follow-up years and treatment	1	334	0.19	0.67
Interaction between follow-up years and P_max_ at baseline (nonlinear 3^rd^ order polynomial expansion)	3	334	2.53	0.057
Interaction between age and sex	1	127	0.46	0.50

In Table 4 are reported the variance parameters with a 95% confidence interval: random effect standard deviation, within-group standard error, and the ratios of the standard deviations between sites 2 and 3 and site 1 (assumed equal to 1 for identification purposes).

**Table 4:** Variance parameters with a 95% confidence interval.

Parameter	Estimate
σ_b_	0.19 (0.16, 0.22)
σ	0.20 (0.17, 0.23)
δ_s2_	0.78 (0.65, 0.94)
δ_s3_	0.89 (0.72, 1.1)

Diagnostic measures were calculated to assess the goodness of the fit of the model. Normal probability plots (not shown) of stratified (by sex, year of follow-up, baseline P_max_, age quartiles, and treatment) within-group standardised residuals, and of the random effects show that the assumptions of normality are reasonable.

Appendix 2

The original data set consisted of 695 longitudinal observations on 139 subjects, with histories of five observations per subject, from baseline to four years. However, the histories were incomplete, with 77 missing P_max_ entries, plus one subject missing all entries for age, sex, treatment and P_max_ measurements from one to four years. After removal of the missing entries, the resulting data set had 613 longitudinal observations; however, the follow-up pattern was not uniform, as shown in Table 5.

**Table 5:** Follow-up patterns with cardinality (number of follow-up occurrences) in the data set. Overall, there were 96 subjects with complete follow-up on all occasions from baseline to four years.

Follow-up pattern	1	01	012	0123	01234	0124
Cardinality	5	3	8	12	96	2
Follow-up pattern	0134	014	0234	03	034	-
Cardinality	1	1	7	2	1	-

The yearly P_max_ measurements split by treatment and site are shown in Figure 4. The graph shows subject variability within each site and variability across sites. Trend slopes don’t seem to change across subjects. These observations informed the choice of model: random effects were used to model the intercept of the patient histories and the site-dependent heteroscedasticity of the P_max_ measurements.
